# An Early Increase of Blood Leukocyte Subsets in Aneurysmal Subarachnoid Hemorrhage Is Predictive of Vasospasm

**DOI:** 10.3389/fneur.2020.587039

**Published:** 2020-12-21

**Authors:** Susanna Bacigaluppi, Federico Ivaldi, Nicola L. Bragazzi, Federica Benvenuto, Fabio Gallo, Alessandro D'Andrea, Paolo Severi, Antonio Uccelli, Gianluigi Zona

**Affiliations:** ^1^Department of Neurosurgery-IRCCS Ospedale Policlinico San Martino, Genoa, Italy; ^2^DINOGMI & CEBR, University of Genoa, Genoa, Italy; ^3^Department of Neurosurgery-E.O. Ospedali Galliera, Genoa, Italy; ^4^DISSAL Department of Health Sciences, Università di Genova, Genoa, Italy

**Keywords:** aneurysmal subarachnoid hemorrhage, inflammatory response, leukocytes, systemic inflammation, immune response, vasospasm

## Abstract

**Objective:** Vasospasm is a severe complication in patients with aneurysmal subarachnoid hemorrhage (aSAH) and cannot be reliably predicted. Its pathophysiology remains elusive with the current body of evidence suggesting inflammation as one of the main driving forces. We here aimed to analyze circulating immune cell subsets over time in patients with aSAH with or without vasospasm.

**Methods:** We performed a prospective observational study recruiting patients with spontaneous aSAH. Peripheral blood withdrawn at pre-specified time-points after aSAH, day 0, days 3–4, 6–8, 10–11, 13–15, and 18–21. Flow cytometry analysis, cell blood counts, and laboratory and diagnostic parameters were performed. Patients were monitored by transcranial Doppler for vasospasm as well as by advanced imaging and divided into a group with (VS) and without vasospasm VS (NVS).

**Results:** We included 42 patients for study analysis, 21 VS and 21 NVS. An early significant increase at day 0 in platelet, leukocyte, neutrophil, lymphocyte, NK lymphocyte, monocyte, and CD 14^++^ CD16^−^ DR^+^ monocyte counts was found in patients with later ensuing vasospasm. The early differences in platelets, leukocytes, lymphocytes, and NK lymphocytes remained significant on multivariate analysis.

**Conclusions:** An early increase of immune cellular subsets in aSAH may contribute to predict VS.

## Introduction

Following aneurysmal subarachnoid hemorrhage (aSAH), cerebral vasospasm is a possible and dreadful condition of narrowing of one or more intracranial arteries. Such vasoconstriction is reflected by a change in flow velocity, detectable by Doppler flowmetry (1). Vasospasm can influence patients' outcome, accounting for additional morbidity and mortality due to ischemia after the primary damage related to the aneurysmal hemorrhage. Its incidence in patients with eaSAH depends on the diagnostic criteria used and on the detection method. Generally, vasospasm has its onset around day 3, the peak incidence around days 6–8, and average reduction around day 12 (2). Vasospasm alone does not account for the pathophysiology of delayed ischemic injury: to a certain degree, vessel narrowing can be compensated by an increased flow velocity; however, perfusion insufficiency can develop. Furthermore, a more distal vasoconstriction may occur such as arteriolar constriction, thrombosis, cortical spreading ischemia, and other processes triggered by early brain injury, which account for delayed ischemic injury (3, 4). It has been observed that outcome in aSAH could relate to the latter. Delayed ischemic injury is a multifactorial result, and proper assessment requires MRI or CT scan (4). When the injury is evident, no reversal is any more possible, thus soliciting the validation of early biomarkers able to predict the onset of vasospasm. Nevertheless, vasospasm remains a well-measurable phenomenon and reflects a local response to arterial damage, arterial blood degradation products, and secondary inflammation. Furthermore, the pathophysiology of vasospasm remains only partially understood: this is reflected by the dearth of drugs useful to improve the clinical outcome in patients with vasospasm, with no therapeutic tool being available yet to avoid the onset of vasospasm itself.

The peripheral inflammatory response following aSAH is characterized by different cellular events such as increased production of cytokines, reactive oxygen species (ROS), and neutrophil activation (5).

Whereas a correlation between inflammatory parameters and clinical outcomes has been demonstrated before (6), the current study aimed to assess thoroughly the possible association between vasospasm, as a local parameter, and a systemic response in terms of cellular subset distribution, in aSAH patients with (VS) or without vasospasm (NVS). In particular, our aim was to assess whether at onset or over time there was a significant difference in blood exams and, in particular, in immune cell distribution between patients that would or not later develop vasospasm after aSAH.

## Patients and Methods

### Study Design and Outline

The protocol for this prospective, observational, multicentric study was approved on May 24, 2013, with the registration number 51/13 by the Ethical Committee of the Polyclinic Hospital San Martino IRCCS, Genoa, Italy, the leading institution for this study, and by the Ethical Committee of the E.O. Ospedali Galliera on March 4, 2013 with the registration number PG 905/13. The present study was carried out in accordance with the Helsinki Declaration of 1975 (revised in 1983).

During that timeframe, the two hospitals managed SAH patients according to the European–American Guidelines for the Management of SAH (7, 8).

At the Policlinico San Martino Hospital IRCCS, Genoa (Italy) and the Galliera Hospital, Genoa (Italy), during the timeframe June 21, 2013 to June 21, 2016, a consecutive series of patients with spontaneous (i.e., non-traumatic) aSAH with admission CT scans within 24 h after aSAH were recruited, after informed consent. This was signed by the patient himself/herself whenever possible or by his/her closest relative where the patient was unable to sign. Diagnosis of intracranial aneurysm was performed by CT angiography (CTA) or, when CTA was either not clear, negative, or whenever required for treatment purposes, by digital subtraction angiography (DSA).

Six time-points were set as follows: day 0, days 3–4, 6–8, 10–11, 13–15, and 18–21 after aSAH. For the first time-point, when a diagnostic DSA was performed after an angio-CT, the first blood sample at arrival was obtained just before the DSA study. At each time-point, blood work included routine exams, and in addition, a sample was collected for lymphocyte flow cytometric characterization.

At each time-point, vasospasm was first monitored by means of transcranial Doppler (TCD), and if the bone window was adequate, three vessels on each side were monitored: the middle cerebral artery (MCA), both distal and proximal sections; the internal carotid artery (ICA)/anterior cerebral artery (ACA); and the posterior communicating artery (PComA)/posterior cerebral artery (PCA). A patient was considered positive for vasospasm if one or more vessels presented increased flow velocity according to well-standardized thresholds (1). Lindegaard ratio, which relates the velocity of the studied intracranial vessel to the velocity of the extracranial internal carotid artery at the neck to differentiate between increased flow velocity due to generalized hemodynamic augmentation and increased velocity due to local vessel narrowing, was not available in all cases. However, since in all cases flow velocity increased only in single vessels, we attributed the cause of such an increase to territorial vessel narrowing rather than to general increased hemodynamics. According to our protocol, in case of inadequate bone window, CTA and/or DSA were used to check for vasospasm in case of neurological deterioration not otherwise explained (1).

Exclusion criteria in study analysis were as follows: death before days 6–8, re-bleeding, moribund patients at admission with coexisting immunological disorders, severe sepsis, and inadequate flow cytometry acquisitions. Further, patients with *sine materia* aSAH were excluded. We aimed at having the most homogenous group as possible in order to have an adequate statistical power for the analysis. Few patients not in homogenous conditions (such as those with re-bleeding, acute severe neurological deterioration, and onset of severe sepsis) would confound the picture and would require a larger group in terms of number of patients for reaching an adequate power analysis threshold.

Data collected included gender, age, history of allergy, cigarette smoking, alcohol abuse, family history positive for aSAH, previous stroke or cerebrovascular accidents, cardiopathy, dyslipidemia, diabetes mellitus, hypertension, use of statins before and/or during hospitalization, consumption of tranexamic acid before aneurysm occlusion, Glasgow Coma Score (GCS), World Federation of Neurosurgical Societies (WFNS) aSAH grading scale, Hunt–Hess and Fisher scores, modality and timing of aneurysm treatment (surgical or endovascular), presence of multiple aneurysms, troponin rise at admission, and insurgence of infective complications. Since the study was observational (it did not interfere with the clinicians' management of SAH patients), and due to the lack of evidence in the guidelines against steroid use, we analyzed for possible differences in steroid administration between the two groups across the study period. Further, variations of neurological status at each time-point were annotated as well, and modified Rankin score (mRS) was marked at the last time-point.

### Laboratory Methods

*Leukocyte Characterization*: Peripheral blood mononuclear cells (PBMC) were prepared on a Ficoll gradient and the pellet was re-suspended with a dilution of 1 × 106 cells/ml in Fluorescence-activated cell sorting (FACS) buffer. Then, 100 μl of suspension were distributed in four tubes:

- Tube 1: not stained, 100 μl PBMC suspension;
- Tube 2: for CD3 assessment: 100 μl PBMC suspension, monoclonal antibodies (3 μl CD3 FITC—BD Bioscience 561806 + 40 μl FACS buffer);
- Tube 3: for T and B cell characterization, 100 μl PBMC suspension, monoclonal antibodies (3 μl CD3 FITC + 3 μl CD4 PE—BD Bioscience 555347 + 2 μl CD8 PE-Cy7—BD Bioscience 557746 + 3 μl CD19 PE-Cy5—BD Bioscience 555414);
- Tube 4: for monocyte (M1 and M2) and natural killer (NK) characterization, 3 μl CD3 FITC + 2 μl CD56 PE-Cy7—BD Bioscience 557747 + 3 μl CD16APC—BD Bioscience 561248 + 2 μl CD14APC-Cy7—BD Bioscience 561384 + 3 μl HLA-Dr PerCP—BD Bioscience 347402).

Incubation for staining was performed at 4°C for 30 min and the tubes were washed with 2 ml FACS buffer, spinned at 1,500 rpm × 5 min, dechanted, and re-suspended in 500 μl of FACS buffer.

The tubes were analyzed with FACSCanto II (BD, USA) and with BD FACSDiva v8.1. The gating strategy for the different subpopulations of leukocytes analyzed by flow cytometry is shown in Supplementary Figure 1.

### Statistical Methods

Before statistical processing and analysis, data were visually inspected for potential outliers in order to see which statistical test was the most adequate for the analysis of the data, based on the distribution of data. Normality of data distribution was checked with the Shapiro–Wilk test. Given the small sample size employed, this test was preferred over other tests, such as the D'Agostino-Pearson *omnibus* test. Univariate analyses (Student's *t* test or its non-parametric test in case of violation of normality of data distribution and chi-squared test) were used to compare the two groups (VS and NVS) for age and gender distributions, comorbidities, risk factors for aSAH, clinical status and radiological scores at presentation, treatment modality, and complications. Given the exploratory nature of the present studies, concerning the different time-points, both uncorrected and corrected *p*-values for multiple comparisons were computed (with significance thresholds of 0.05 and 0.008, respectively). Regarding the multiple comparisons issue, both uncorrected and corrected *p*-values were calculated. On the one hand, conducting multiple tests increases the overall type I error rate and on the other hand, adjusting significantly inflates the type II error rate. Moreover, it is still controversial: (i) which correction test to apply and (ii) the number of adjustments that should be undertaken (9). In the present study, both conservative (Bonferroni's) and less-conservative (Holm's) methods were utilized. Multivariate logistic regression analysis (conducted on those variables significant at the univariate analysis and adjusting for confounding factors such as Fisher and Hunt–Hess scores and WFNS) was conducted to shed light on the determinants of VS in aSAH patients. Moreover, receiver operating characteristic (ROC) analysis was performed in order to investigate whether cellular immunological parameters could discriminate according to the vasospasm status. For this purpose, the area under the curve (AUC) was computed for those parameters significant at the univariate analysis as variables and VS status as classification variable. Furthermore, the Youden's *J* index was used to determine the best cut-off point in order to achieve acceptable sensitivity and specificity levels.

Using the GPower software for calculating the *a priori* sample size power, for repeated measurement analysis, assuming an alpha error probability of 0.05, a power of 0.80, and two independent groups (VS and NVS), with six different time-points, and no information about correlations among the serial measurements, expecting a small-to-medium effect size based on the existing scholarly literature (10, 11), the minimum sample size requested was found to be 36.

*Post-hoc* power analysis of the sample size demonstrated statistical robustness of our study.

GraphPad Prism Software v7, “Statistical Package for Social Sciences” (SPSS v24 for Windows), and MedCalc Statistical Software version 17.9.7 (MedCalc Software bvba, Ostend, Belgium; http://www.medcalc.org; 2017) were used for statistical analyses. Figures with *p*-value <0.05 were considered statistically significant.

## Results

### Baseline Characteristics of Study Population

**Figure 1:**
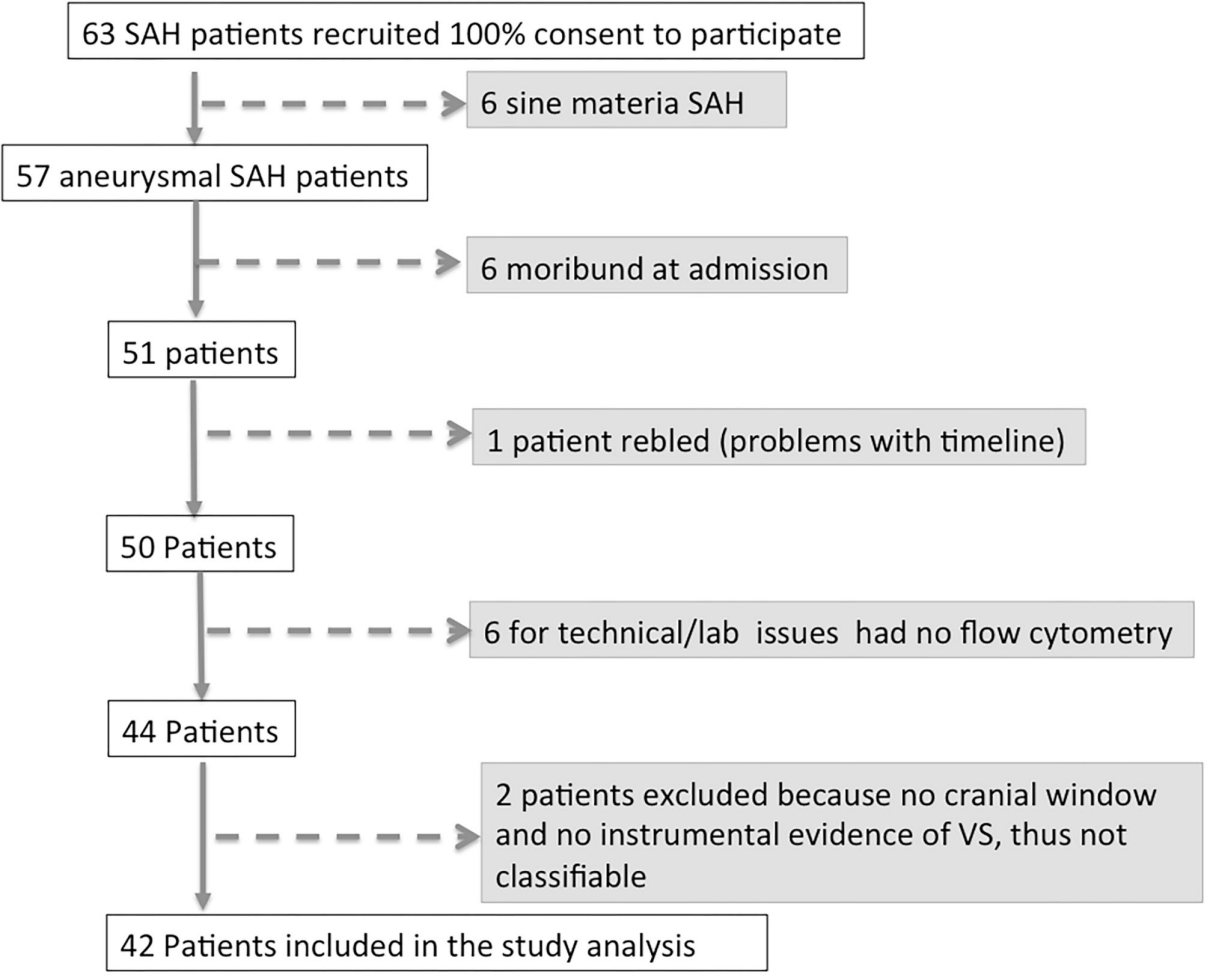
Flow chart of patients in the study.

**Figure 2:**
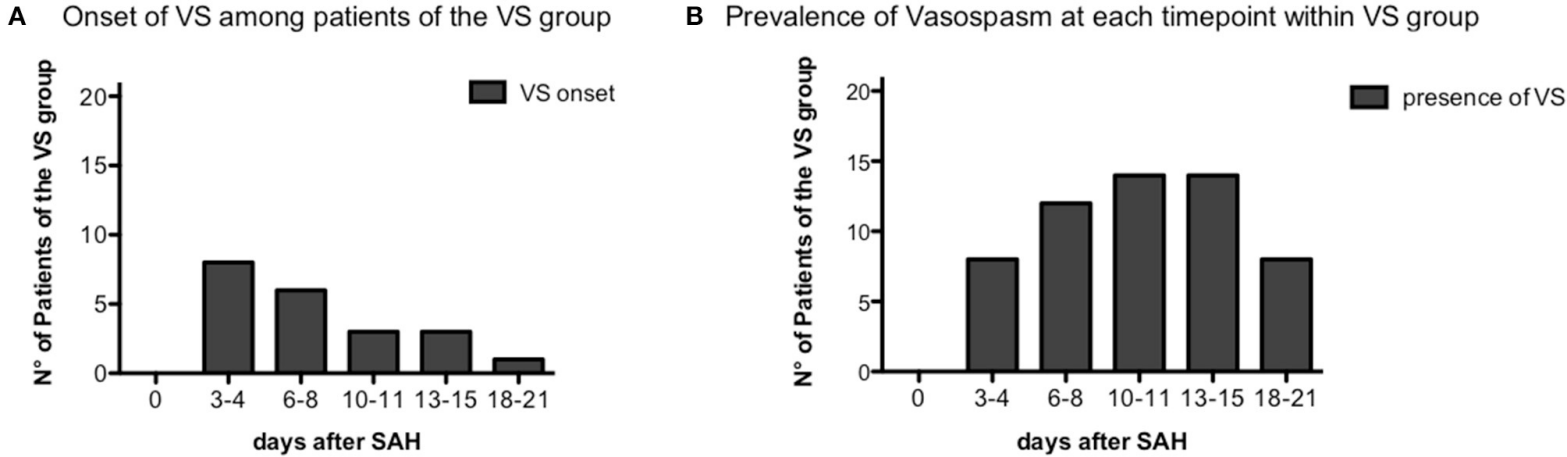
Vasospasm onset time. **(A)** Distribution of vasospasm onset over the different time-points among the vasospasm (VS) patient group, *n* = 21. **(B)** Prevalence of vasospasm over the time-points of the study among VS patients, *n* = 21.

Between June 21, 2013 and June 21, 2016, 63 patients affected by aSAH were recruited with a 100% rate of study participation. Subsequently, 15 of 63 patients (33.3%) were excluded from the analysis, since six patients had a definitive diagnosis of *sine materia* aSAH, six patients were moribund at admission (i.e., unsalvageable patients), and one patient re-bled. Of the remaining 50 patients, in six cases, for technical issues, it was not possible to perform flow cytometry analysis, and thus, they were excluded from the analysis. Two more patients were excluded from the analysis, being unclassifiable for the present study purpose having neither cranial window for TCD nor any instrumental exam documenting vasospasm. Forty-two patients with aSAH were thus included in the study (see flowchart on Figure 1). On the days following aSAH, 21 patients developed vasospasm (VS group) while 21 patients did not present vasospasm (NVS group). In the NVS group, all included patients had a cranial window for Doppler diagnostics, and in the VS group, there were three patients with no cranial window. In these patients, angiography and/or CTA confirmed the suspicion of VS. None of the aSAH patients in this study showed vasospasm at day 0: in most cases, vasospasm onset was detected with transcranial Doppler (TCD) at days 3–4 after aSAH (38.1%) or on days 6–8 (28.6%). Only 14.3% patients became positive at days 10–11, 15% at days 13–15, and 4.7% at days 18–21 (Figure 2A). The prevalence of vasospasm among these 21 patients was highest between days 6 and 15 (Figure 2B).

At admission, the two groups did not statistically differ for gender distribution (male/female ratio of 1:1.4 and 1:3.4 for the NVS and VS groups, respectively) and age (59.1 ± 15.05 for the NVS group vs. 57.43 ± 15.41 for the VS group). Further, there were no differences in history of cigarette smoking, allergy, and comorbidities, including hypertension, cardiopathy, previous stroke, and dyslipidemia. Also, the prevalence of multiple aneurysms, family history of aSAH or presumed warning leaks did not significantly differ between the two groups. Fisher score was different (2.43 ± 1.21 in the NVS group vs. 3.14 ± 0.96 in the VS group, *p* = 0.0356), as well as WFNS (1.57 ± 1.12 in the NVS group vs. 2.286 ± 1.15 in the VS group, *p* = 0.036) and Hunt–Hess scale (1.86 ± 1.06 in the NVS group vs. 2.57 ± 1.12 in the VS group, *p* = 0.021), while the GCS scale did not differ between the groups (14 ± 2.41 in the NVS group vs. 13.1 ± 2.68 in the VS group, *p* = 0.26) (Table 1).

**Table 1:** Characteristics of the 42 patients included in the study and of treatment modality.

	**NVS (21 patients)**	**VS (21 patients)**	***P*-value**
Gender: M; F	9; 12	4; 17	0.18
Age (mean, SD)	59.1 ± 15.05	57.43 ± 15.4	*t* test unpaired, 2-tailed 0.72
Allergy	3/21	4/20	0.7
History of smoking	11/20	11/19	1.00
Hypertension	13/21	11/21	0.76
Cardiopathy	1/21	1/21	1
Previous stroke	1/21	2/21	1.00
Dyslipidemia	12/18	10/18	0.73
Multiple aneurysms	5/22	8/21	0.33
Family history for SAH	1/20	3/20	0.61
Warning leaks	2/21	6/21	0.24
Fisher scale (mean, SD)	2.43 ± 1.21	3.14 ± 0.96	*t* test unpaired, 2-tailed 0.0403(*)
I	7	2	
II	3	2	
III	6	8	
IV	5	9	
GCS (mean, SD)	14 ± 2.41	13.1 ± 2.68	*t* test unpaired, 2-tailed 0.26
WFNS (mean, SD)	1.57 ± 1.12	2.29 ± 1.15	*t* test unpaired, 2-tailed 0.0479(*)
I	15	6	
II	3	8	
III	1	2	
IV	1	5	
V	1	0	
Hunt–Hess (mean, SD) *n* (NVS) = 21; *n* (VS) = 21	1.86 ± 1.06	2.57 ± 1.12	*t* test unpaired, 2-tailed 0.0403(*)
I	9	3	
II	9	9	
III	1	4	
IV	1	4	
V	1	1	
**Treatment modality**			
Surgical/endovascular	2; 19	6; 15	0.24
Hematoma evacuation	1/21	4/21	0.34
External ventricular drainage	6/20	9/21	0.52
Tranex before treatment	6/21	9/21	0.52
Statins before SAH	2/20	1/21	0.61
Statins after SAH	4/21	1/21	0.18
Steroids	8/21	15/21	0.07
Infection	8/21	11/21	0.54
Positive blood culture	3/21	2/21	1.00
Ischemia (CT findings)	5/21	10/21	0.2
Outcome as mRS			*t* test unpaired, 2-tailed 0.0529
0	7/21	3/21	
1	5/21	4/21	
2	2/21	1/21	
3	0/21	1/21	
4	5/21	6/21	
5	2/21	5/21	
6	0	1/21	

Significance was assessed with chi square test, where not specified. *p-value < 0.05.

Treatment modality intended as surgical vs. endovascular aneurysm occlusion with or without hematoma evacuation as with or without a ventricular drainage was again comparable and not statistically different between the two patient groups (Table 1).

The analysis of the administration of specific drugs intended as ever administered or not (tranexamic acid before aneurysm occlusion rather than steroids during hospitalization or statins before and/or after hospitalization) did not reach statistical significance between the two groups (Table 1). However, in more detail, at day 0, none of the patients received steroids, but a difference was noted for the time-points days 10–11 (*p* = 0.006) and 13–14 (*p* = 0.006) with a greater use of steroids in patients belonging to the VS group.

Concerning complications during the observed timeframe (days 0–21 after SAH), the incidence of any infection (e.g., pneumonia or urinary tract infection) was also recorded. The positivity at CT scan for hypodensities indicating delayed ischemia of various origins not detectable at admission was also comparable and not statistically significantly different between the two groups as well (Table 1).

The present study's primary aim was not to evaluate the patient outcome. We nonetheless registered at the last study time-point 18–21 days and for some patients discharged at the fifth time-point the modified Rankin scale (mRS) (see Table 1).

There were no significant correlations between mRS and the occurrence of vasospasm after SAH. mRS did correlate with the presence of ischemic lesions at CT scan (*p* = 0.0034). There was no correlation between VS and ischemic lesions at CT scan. In our study cohort, there was no correlation between VS and new neurological alterations.

### Blood Profile

**Figure 3:**
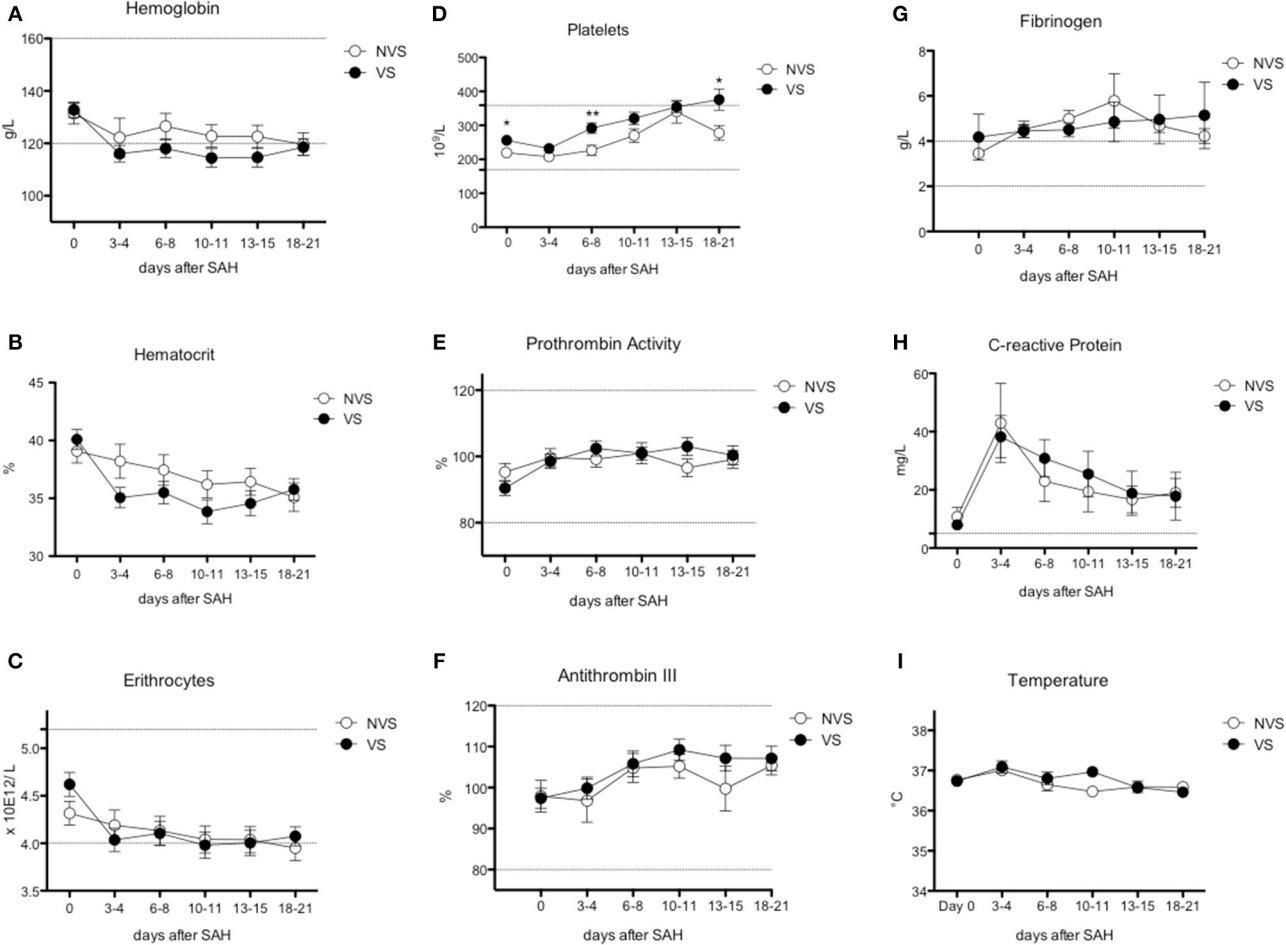
Erythrocyte, platelet, and inflammatory markers over time after aSAH. **(A)** Hemoglobin (Hb); **(B)** hematocrit; **(C)** erythrocytes; **(D)** platelets; **(E)** prothrombin activity; **(F)** antithrombin III; **(G)** fibrinogen; **(H)** C-reactive protein; **(I)** temperature. Graphs showing mean values of patients, *n* = 42, at the six time-points defined as days after SAH. The vertical bars show mean and standard error. Non-vasospasm group (NVS), *n* = 21, in white circles and the vasospasm group (VS), *n* = 21, in black circles. The stars (*) indicate statistical significance at the unpaired two tailed *t* test made at day 0. Small dotted horizontal lines indicate in the graph the normal range reference values where available and or useful.

**Figure 4:**
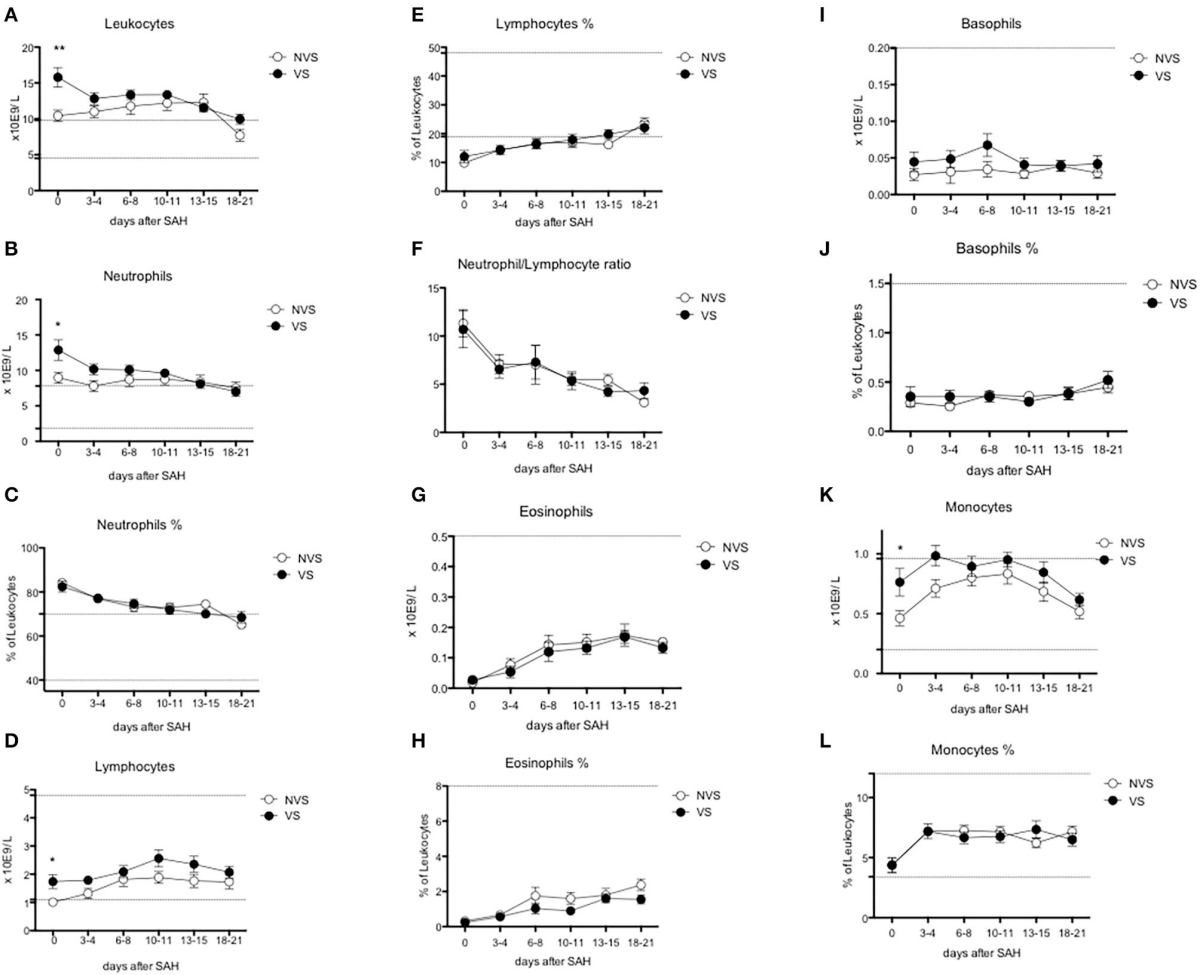
Circulating leukocyte subpopulations over time after aSAH. **(A)** Leukocytes; **(B)** neutrophils; **(C)** % of neutrophils; **(D)** lymphocytes; **(E)** % of lymphocytes; **(F)** neutrophil/lymphocyte ratio; **(G)** eosinophils; **(H)** % of eosinophils; **(I)** basophils; **(J)** % of basophils; **(K)** monocytes; **(L)** % of monocytes. Graphs showing mean values of patients, *n* = 42, at the six time-points defined as days after SAH. The vertical bars show mean and standard error. Non-vasospasm group (NVS), *n* = 21, in white circles and the vasospasm group (VS), *n* = 21, in black circles. The stars (*) indicate statistical significance at the unpaired two tailed *t* test made at day 0. Small dotted horizontal lines indicate in the graph the normal range reference values where available and or useful.

**Figure 5:**
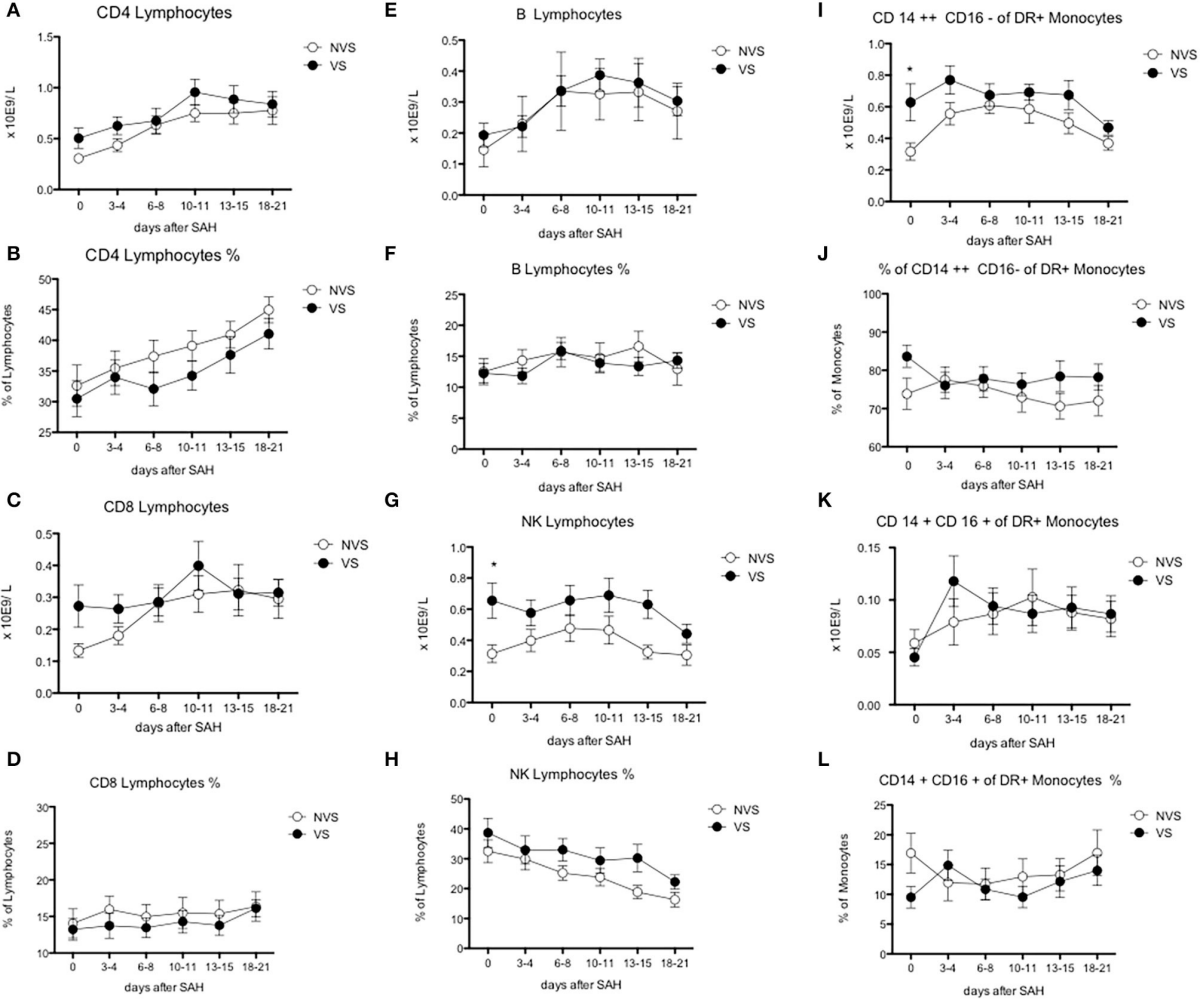
Blood lymphocyte subsets and monocyte counts after aSAH. **(A)** CD4-positive lymphocytes; **(B)** % of CD4-positive lymphocytes; **(C)** CD8-positive lymphocytes; **(D)** % of CD8-positive lymphocytes; **(E)** B lymphocytes; **(F)** % of B lymphocytes; **(G)** natural killer (NK) lymphocytes; **(H)** % of natural killer (NK) lymphocytes; **(I)** CD CD14++ CD16– of DR+ monocytes; **(J)** % of CD14++ CD16– monocytes; **(K)** 14+ CD16+ of DR+ monocytes; **(L)** % of CD14+ CD16+ of DR+ monocytes. Graphs showing mean values of patients, *n* = 42, at the six time-points defined as days after SAH. The vertical bars show mean and standard error. Non-vasospasm group (NVS), *n* = 21, in white circles and the vasospasm group (VS), *n* = 21, in black circles. The stars (*) indicate statistical significance at the unpaired two tailed *t* test made at day 0. Small dotted horizontal lines indicate in the graph the normal range reference values where available and or useful.

The trend of blood exams and circulating cells was analyzed (see Figures 3–5), and at day 0, the two groups (VS vs. NVS) were analyzed for significant differences.

**Figure 6:**
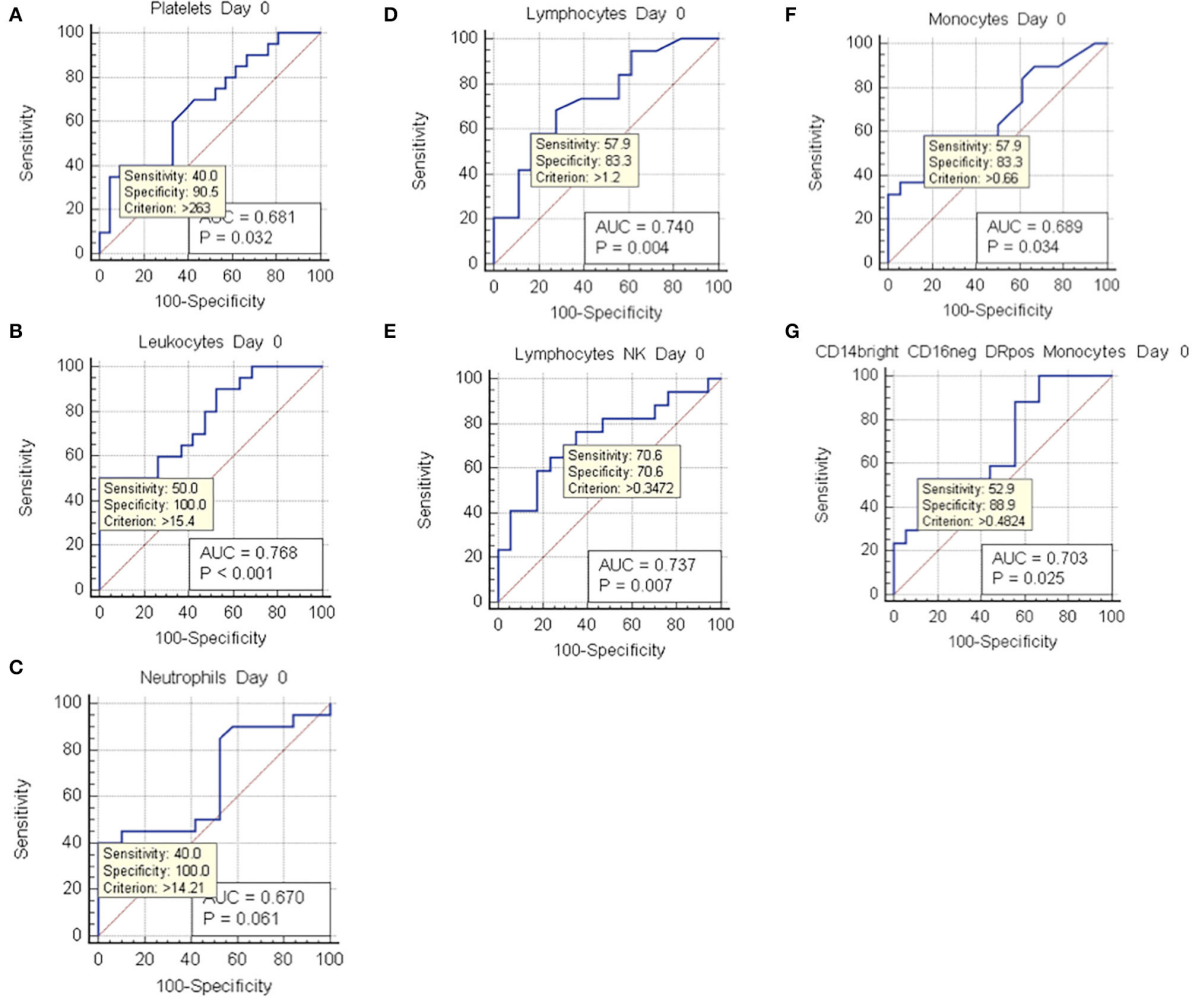
ROC curve analysis at day 0. ROC curves of values with positive two-tailed *t* test at day 0, which showed statistically significant differences between non-vasospasm (NVS), *n* = 21, and vasospasm (VS) patients, *n* = 21). Sensitivity and specificity for possible cut-off values (criterion) and *p* values are shown besides the area under the curve (AOC). **(A)** Platelets; **(B)** leukocytes; **(C)** neutrophils; **(D)** lymphocytes; **(E)** natural killer (NK) lymphocytes; **(F)** monocytes; **(G)** CD14++ CD16– DR+ monocytes.

Results of the two-tailed Student's *t* test are reported in Table 2. For significant values, a further analysis was performed correcting for Fisher and Hunt–Hess scores and WFNS, which were significantly different between the two groups at baseline (see Table 3). Further, for values statistically significant at day 0 between the two groups, ROC curve analysis was performed to identify possible cut-off values, together with their statistical sensitivity, specificity, and significance value (see Figure 6).

**Table 2:** Values at day 0 followed over time in VS and NVS patients, respectively.

**Measures at day 0**	**NVS (21 patients)**	**VS (21 patients)**	***P*-value**
Hemoglobin (g/L)	131.41 (17.27)	132.8 (13.03)	0.7776
Hematocrit (%)	39.07 (4.44)	40.09 (3.8)	0.4446
Erythrocytes (×10^9^/L)	4.32 (0.54)	4.62 (0.56)	0.0931
Platelets (×10^9^/L)	219.33 (52.12)	256.1 (52.12)	**0.0296**
Prothrombin activity (%)	95.28 (11.07)	90.4 (9.81)	0.1585
Antithrombin III (%)	97.88 (15.91)	97.42 (10.77)	0.9187
Fibrinogen (g/L)	3.46 (0.83)	4.17 (4.56)	0.5028
C-reactive protein (mg/L)	10.76 (14.12)	7.96 (6.9)	0.4424
Temperature (°C)	36.76 (0.45)	36.74 (0.35)	0.8366
Leukocytes (×10^9^/L)	10.43 (3.45)	15.79 (6.07)	**0.0018**
Neutrophils (×10^9^/L)	8.98 (3.4)	12.87 (6.43)	**0.0247**
Neutrophils (%)	84.17 (7.66)	82.42 (10.83)	0.5670
Lymphocytes (×10^9^/L)	1.01 (0.57)	1.74 (1.08)	**0.0150**
Lymphocytes (%)	9.86 (5.7)	12.07 (9.36)	0.3947
Neutrophil-to-lymphocyte ratio	11.32 (6.12)	10.68 (8.28)	0.7894
Monocytes (×10^9^/L)	0.46 (0.27)	0.76 (0.5)	**0.0295**
Monocytes (%)	4.35 (2.63)	4.39 (2.67)	0.9594
Eosinophils (×10^9^/L)	0.02 (0.03)	0.03 (0.04)	0.5052
Eosinophils (%)	0.33 (0.52)	0.24 (0.37)	0.5454
Basophils (×10^9^/L)	0.03 (0.03)	0.04 (0.06)	0.2789
Basophils (%)	0.29 (0.17)	0.35 (0.43)	0.5572
Lymphocyte CD4 (×10^9^/L)	0.31 (0.17)	0.5 (0.42)	0.0808
Lymphocyte CD4 (%)	32.62 (13.91)	30.49 (12.04)	0.6364
Lymphocyte CD8	0.13 (0.09)	0.27 (0.27)	0.0544
Lymphocyte CD8%	14.05 (8.26)	13.24 (6.15)	0.7454
Lymphocyte B	0.15 (0.22)	0.19 (0.16)	0.4757
Lymphocyte B%	12.51 (8.69)	12.26 (6.54)	0.9227
NK lymphocytes (×10^9^/L)	0.31 (0.23)	0.66 (0.47)	**0.0109**
NK lymphocytes %	32.53 (15.8)	38.7 (20.05)	0.3264
CD14++ CD16– DR+ monocytes (×10^9^/L)	0.32 (0.23)	0.63 (0.49)	**0.0199**
CD14++ CD16– DR+ monocytes (%)	73.85 (16.76)	83.62 (11.93)	0.0636
CD14+ CD16+ DR+ monocytes (×10^9^/L)	0.06 (0.05)	0.05 (0.03)	0.3863
CD14+ CD16+ DR+ monocytes (%)	16.93 (13.29)	9.51 (6.93)	0.0636

*Mean values and SD are presented and results of univariate analysis (two-tailed t test), with uncorrected p-value, significant if < 0.05. Bold values indicate statistically significant differences p < 0.05*.

**Table 3:** Multivariate logistic regression analysis of values at day 0 (those followed over time) with correction for Fisher grade, WFNS, and Hunt–Hess score of patients with and without vasospasm.

**Variable**	**Coefficient**	**Std. Error**	**Wald**	***P***	**Odds ratio**	**95% CI**
Platelets	0.02	0.01	5.29	**0.0214**	1.02	1.00 to 1.03
Leukocytes	0.26	0.11	5.92	**0.0150**	1.29	1.05 to 1.59
Neutrophils	0.16	0.08	3.64	0.0565	1.17	1.00 to 1.38
Lymphocytes	1.19	0.60	3.87	**0.0493**	3.27	1.00 to 10.68
NK lymphocytes	2.99	1.53	3.81	**0.0509**	19.84	0.99 to 398.12
Monocytes	1.93	1.15	2.82	0.0930	6.88	0.72 to 65.37
CD14++ CD16– DR+ Monocytes	−10.79	11.02	0.96	0.3272	0.00	–

*Bold values indicate statistically significant differences (p ≤ 0.05)*.

Hemoglobin and hematocrit, red blood cell count, platelets, prothrombin activity, and anti-thrombin III were not significantly different between the two groups (Figures 3A–F). Fibrinogen values tended to stay above the upper limit of normality at all the time-points in both groups (Figure 3G). C-reactive protein (CRP) did not present a statistically significant difference between the two groups. Nevertheless, CRP trend presented a peak at days 3–4 with progressive decrease toward a plateau at days 13–15 (Figure 3H). Temperature was comparable in both groups with only a small difference at days 10–11 (Figure 3I). Platelets showed an increasing trend, still remaining within normal reference values, with all the mean values being higher in VS patients, and significantly higher at days 0, 6–8, and 18–21 (Figure 3D). At the ROC analysis, platelet values differed among VS and NVS groups with a sensitivity of 40% and a specificity of 90.5% (cut-off > 263 × 10^9^/L) (AUC = 0.668, *p* = 0.032) (Figure 6A).

### White Blood Cells

Leukocyte values were significantly increased at day 0 in the VS patients (15.79 ± 1.36 × 10^9^/L vs. 10.43 ± 0.79 × 10^9^/L, *p* = 0.0018) (Figure 4A). The levels were above the upper limit in both groups and tended to normalize toward the last time-point at 3 weeks after SAH (days 18–21). At the ROC analysis, leukocyte values differed among VS and NVS groups with a sensitivity of 50% and a specificity of 100% (cut-off >15.4 × 10^9^/L) (AUC = 0.788, *p* < 0.001) (Figure 6B).

### Blood Profile and White Blood Cells

Since at the ROC analysis the stronger associations with VS were found for high platelet and leukocyte counts at day 0, even though with low sensitivity values, we investigated whether their combination would increase sensitivity. We were able to compute a sensitivity of 60.0%, a specificity of 89.5%, and an AUC of 0.771 (*p* = 0.0004).

### Neutrophils

Both groups presented an increased percentage of neutrophils at day 0, which then progressively decreased over time. Moreover, the absolute number of neutrophils was significantly higher among VS patients at day 0 (12.87 ± 1.44 × 10^9^/L vs. 8.98 ± 0.78 × 10^9^/L, *p* = 0.0247) (Figures 4B,C). At the ROC analysis, absolute neutrophil number differed among VS and NVS groups with a sensitivity of 40% and a specificity of 100% (cut-off value >14.21 × 10^9^/L, AUC = 0.61, *p* = 0.061) (Figure 6C).

### Lymphocytes and Lymphocyte Subpopulations

At day 0, absolute lymphocyte count was significantly higher in VS patients (1.74 ± 0.25 × 10^9^/L vs. 1.01 ± 0.13 × 10^9^/L, *p* = 0.015). Lymphocyte percentage was initially below the normal range and tended to increase over the time (Figures 4D,E). At the ROC analysis, lymphocyte absolute number differed according to the vasospasm status with a sensitivity of 57.9% and a specificity of 83.3% (cut-off value >1.2 × 10^9^/L, AUC = 0.74, *p* = 0.004) (Figure 6D). The Neutrophil/Lymphocyte ratio showed a decreasing trend after day 0 with no significant differences between VS and NVS patients (Figure 4F).

Regarding lymphocyte subgroups, we analyzed by flow cytometry CD4-, CD8-, T-, B-lymphocyte and could not detect significant differences at all time-points between the two study groups (Figures 5A–F).

Interestingly, NK lymphocyte absolute count was higher, at day 0 in VS patients (0.656 ± 0.11 × 10^9^/L in the VS vs. 0.31 ± 0.06 × 10^9^/L in the NVS patients, *p* = 0.0109, even though not significant after adjusting for multiple comparisons). At the ROC analysis, NK values differed among the VS and NVS groups with a sensitivity of 70.6% and a specificity of 70.6% (AUC = 0.737, *p* = 0.007) (Figure 6E).

### Eosinophils, Basophils, and Monocytes

Eosinophils and basophils both in terms of absolute numbers and percentages remained constant within normal ranges and were not different between the two groups (Figures 4G–J), except for a small, borderline difference in eosinophils at 18–21 days (two-tailed Student's *t* test 1.55 ± 0.25 in the VS vs. 2.38 ± 0.33 in the NVS patients, *p* = 0.05).

Regarding monocytes, a statistically significant difference of the absolute number was found at day 0 in VS patients (0.76 ± 0.11 × 10^9^/L in the VS vs. 0.46 ± 0.06 × 10^9^/L in the NVS patients, *p* = 0.029) (Figures 4K,L).

At the ROC analysis, monocyte absolute numbers differed among VS and NVS patients with a sensitivity of 57.9% and a specificity of 83.3% (cut-off value >0.66 × 10^9^/L) (AUC = 0.68936, *p* = 0.034) (Figure 6F).

Monocytes were characterized more in detail by means of flow cytometry (Figures 5I–L) in order to be able to capture the classical activated M1 monocytes (CD14+ CD16+ DR+) and the alternative activated M2 monocytes (CD14++ CD16– DR+) responses, which have been described to have different effects in patients with stroke (12, 13). CD14++ CD16– DR+ monocytes were significantly higher at the two-tailed Student's *t* test in VS patients at day 0 (0.6 ± 0.12 × 10^9^/L in the VS vs. 0.32 ± 0.05 × 10^9^/L in the NVS patients, *p* = 0.02). At the ROC analysis, CD14++ CD16– DR+ monocyte number differed among the VS and NVS groups with a sensitivity of 52.9% and a specificity of 88.9% (cut-off value >0.48^9^/L) (AUC = 0.7, *p* = 0.025) (Figure 6G).

### Re-analysis Correcting for Multiple Comparisons

When adjusting for multiple testing, only leukocyte count remained statistically significant, either applying the conservative (Bonferroni's) or the less-conservative (Holm's) correction technique.

### Re-analysis Correcting for Baseline Differences

Among values measured over the time, which were significant at day 0 at the two-tailed Student's *t* test, the following were positive at a re-analysis corrected for Fisher and Hunt–Hess scores and WFNS: platelets (*p* = 0.02), leukocytes (*p* = 0.015), lymphocytes (*p* = 0.0493), and NK lymphocytes (*p* = 0.0509). On the contrary, the following values analyzed after correction at day 0 were not significant: neutrophils (*p* = 0.057), monocytes (*p* = 0.093), and CD14++ CD16– DR+ monocytes (*p* = 0.327) (Table 3).

As concerns possible differences in leukocyte subgroups between patients with and without steroids, we have noted a significant difference of steroid administration at time-points 10–11 days and 13–15 days with more patients in the VS group. However, when re-analyzing all leukocyte subgroups, no differences were found for these time-points in patients with or without steroids. The choice to use steroids neither correlated to ischemia nor to grade of ischemia at CT scan nor Fisher score at arrival.

## Discussion

This study aimed to identify possible differences between VS and NVS patients in terms of immune cellular profile at various time-points and up to 3 weeks after aSAH.

The role of platelets in post-aSAH vasospasm has been put forth in the theory of platelet activation and aggregation, with high expression of platelet surface receptor glycoprotein GpIIb/IIIa and impairment of the anti-adhesive function of endothelium (14). These alterations contribute to the insurgence of worse consequences and adverse events after vasospasm, with a poorer prognosis. A decreasing platelet count at days 3–4 compared to that at admission and a subsequent increase until days 19–20 was observed by other authors as well (13). As in the present study based on TCD, other authors noticed a higher platelet count in aSAH patients with delayed cerebral ischemia (DCI) (15).

Our study pointed a possible cut-off value for platelets for increased vasospasm risk of 263 × 10^9^/L with a high specificity (90%), despite a modest sensitivity (40%), which means that platelet value alone does not capture all patients at risk. The role of platelets in VS patients is demonstrated by some trials showing that the use of dual anti-platelet therapy (clopidogrel and aspirin) can be effective in reducing risk of clinical vasospasm and DCI (14).

As other authors, we observed an increase in CRP values at days 3–4. For instance, among 100 aSAH patients, an increase in CRP level from the admission to day 3 was reported, followed by a decrease until day 9 (11). In another study, CRP levels peaked on day 3, with statistically significant differences between the VS and the NVS groups on days 1, 3, and 5 (10). Statistically significant differences concerning CRP levels in the first 24 h after aSAH have been described in a sample of 57 patients without and 21 patients with vasospasm (13.06 ± 20.18 vs. 36.31 ± 59.86 pg/ml, respectively) (16). In a sample of 93 aSAH patients, CRP was found to be a useful biomarker of clinical outcomes (17). In some studies, this increase has been shown to last until days 6–8, correlating with poorer neurological outcomes. For instance, an increase in mean CRP level has been detected in aSAH patients, with a steady increase until day 6 (18). In a cohort of 88 aSAH patients, CRP level was higher on days 5, 6, 7, and 8 in the group developing delayed ischemic neurological deficit (18).

We further observed an early and significant increase in leukocytes in aSAH individuals with vasospasm compared to those without, which remained above the upper limits until days 18–20. Furthermore, at day 0, the difference was statistically significant. The ROC curve analysis showed a possible cut-off value of 15.4 × 10^9^/L, with a very high specificity (100%) despite a modest sensitivity (50%). Leukocytosis has been well-recognized as a risk factor for vasospasm. In a retrospective study on 224 patients treated for aSAH, a leukocyte count peak >15 × 10^9^/L remained independently associated with symptomatic vasospasm. Moreover, the greatest difference between patients with and without vasospasm had occurred 3 days after aSAH (19). Similarly, in a retrospective study conducted in a sample of 542 consecutive aSAH patients, leukocyte count ≥11 × 10^9^/L on admission was a predictor of the insurgence of vasospasm (20). Leukocyte count was found to closely match with occurrence of DCI in a prospective study (21).

Regarding the role of neutrophils, we observed an early statistically significant difference in neutrophils between VS and NVS patients. This has also been consistently noted in a sample of 55 consecutive aSAH patients (22). More in detail, a concentration of neutrophils at day 0 over 14.21 × 10^9^/L showed a high specificity despite a modest sensitivity in predicting the occurrence of vasospasm. While we found in aSAH an overall early increase of the neutrophil-to-lymphocyte ratio (NLR) similar to other acute vascular disorders (23); interestingly, patients with later ensuing vasospasm did not show a further elevation of the NLR.

Focusing on CD4^+^ and CD8^+^ cells, we found a tendency to increase in the VS group at days 6–8 and 10–11 (although not significant), whereas other authors could not detect any statistically significant difference between VS and NVS patients (16). Some authors found that CD4^+^/CD8^+^ ratio may be involved in the onset of aSAH and of delayed vasospasm (24). Concerning NK cells, other authors detected activated cytotoxic CD16^bright^ CD56^dim^ NK cells in the cerebrospinal fluid of aSAH patients with differences possibly indicating their association with the development of vasospasm and DCI (25). Furthermore, we showed that at day 0, monocytes significantly differed between VS and NVS patients. Regarding M1 and M2 monocytes, our study confirmed their different prognostic role in neuro-inflammation and in stroke (12, 26, 27). In stroke, CD14^++^ CD16^−^ DR^+^ monocytes were also higher in patients with systemic axonal injury, early worsening, poor prognosis, and early mortality, indicating that monitoring of monocyte subtypes may represent a useful tool for predicting clinical course and prognosis in stroke patients (26).

In summary, the specific analysis for significant values at day 0 evidenced differences in platelets, leukocytes, neutrophils, lymphocytes, NK lymphocytes, monocytes, and CD14^++^ CD16^−^ DR^+^ monocytes.

A further multivariate analysis of blood values at day 0 correcting for significant differences at presentation between VS and NVS patients (Fisher grade, WFNS, and Hunt–Hess) showed that neutrophils, monocytes, and CD14^++^ CD16^−^ DR^+^ monocytes failed to remain significantly different between the two groups. This could possibly indicate that differences in the amount of blood in aSAH could associate with different recruitment of those cells. On the contrary, the other blood values (platelets, leukocytes, lymphocytes, and NK lymphocytes) that were confirmed to be statistically significantly different after correction may have an additional role in the pathogenesis and possible prediction of vasospasm.

To the best of our knowledge, at present, this study is one of the few reports in literature that has comprehensively characterized systemic inflammatory cellular response in aSAH patients in relation to vasospasm. Strengths include a longitudinal, prospective, multicentre design, whereas the major shortcoming is given by the small sample size employed, even though still adequate for an exploratory analysis in line with the results of the power calculation.

In conclusion this preliminary study confirms that early changes of specific immune populations could associate with vasospasm and it could help to identify individuals at risk of developing vasospasm following aSAH. Due to the above-mentioned limitations, future studies should replicate our findings, recruiting larger sample sizes. These could be cohort investigations, enrolling patients based on categories found in our (high vs. low platelets and leukocyte count at admission) and/or other studies and evaluating how many of them will develop VS in the two cohorts.

## Data Availability Statement

The raw data supporting the conclusions of this article will be made available by the authors, upon reasonable request.

## Ethics Statement

The protocol for this prospective, observational, multicentric study was approved on 24 May 2013, with the registration number 51/13 by the Ethical Committee of the Polyclinic Hospital San Martino IRCCS, Genoa, Italy, leading institution for this study and by the Ethical Committee of the E. O. Ospedali Galliera on 4th March 2013 with the registration number PG 905/13. The patients/participants provided their written informed consent to participate in this study.

## Author Contributions

SB is the principal investigator of the study and did the study design, ethics committee request form, data collection, preparation and analysis of great part of blood samples, performance of great part of TCD studies, data analysis, main draft of the manuscript, and approval of the final version. FI contributed in the approval of the study design, set-up of the laboratory procedures for the present study, set-up of the flow cytometry protocol, preparation of blood samples, flow cytometry analysis, and approval of the final manuscript. NB contributed in the approval of the study design, revision of data analysis, re-analysis of data positive at *t* test with correction for baseline differences between the two groups (Fisher, WFNS, and Hunt–Hess), correction of the manuscript, and approval of the final manuscript. FB contributed in the approval of the study design, set-up of the laboratory procedures for the present study, set-up of the flow cytometry protocol, flow cytometry analysis, and approval of the final manuscript. FG contributed in the discussion of several statistical alternative methods and approval of the final manuscript. AD'A contributed in the approval of the study design, support in patient recruitment, and approval of the final manuscript. PS is the Clinical Director for the study collaboration of Galliera Hospital and contributed in the approval of the final manuscript. AU is the Scientific Director of the study and contributed in the approval of the study design, methodological advising, manuscript correction and approval of the final version, and also also hosted our experimental part in his lab with supply of materials, instruments, and support of research staff. GZ the Clinical Director of the study, contributed in the study design approval, supported in the application of the clinical part of the study protocol, granted the ethics committee approval request requested and granted clinical staff collaboration for regular clinical, diagnostic, and blood sample preparation for patient management and for the clinical observational study, and corrected the manuscript and approved of the final version. All authors contributed to the article and approved the submitted version.

## Conflict of Interest

The authors declare that the research was conducted in the absence of any commercial or financial relationships that could be construed as a potential conflict of interest.

## Supplementary Material

Supplementary Figure 1 Flow cytometry gating strategy. We here illustrate the applied gating strategy for considered monocyte **(C,D)** and lymphocyte **(E–H)** subpopulations. **(A)** Selection of alive cells after Ficoll gradient; **(B)** morphological identification of monocytes and lymphocytes; **(C)** HLA DR+ monocytes; **(D)** CD14+ CD16+ and CD 14++ CD16− HLA DR+ monocytes; **(E)** T-helper (CD4+) lymphocytes; **(F)** cytotoxic (CD8+) lymphocytes; **(G)** B lymphocytes; **(H)** natural killer (NK) lymphocytes.
