# Supplementary figures and images for: An Early Increase of Blood Leukocyte Subsets in Aneurysmal Subarachnoid Hemorrhage Is Predictive of Vasospasm
Source: Front Neurol. 2020 Dec 21;11:587039. doi: 10.3389/fneur.2020.587039 (PMC7779675; doi:10.3389/fneur.2020.587039)

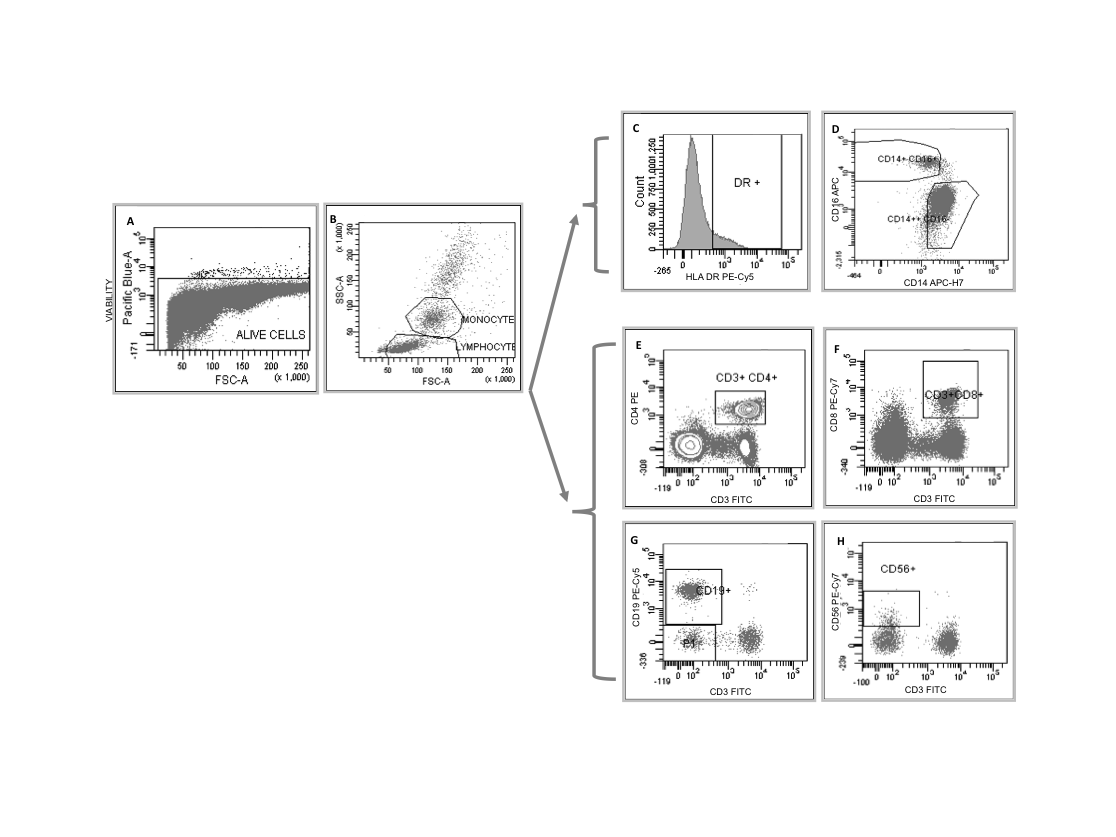

Supplement: Supplementary Figure 1 — Flow cytometry gating strategy. We here illustrate the applied gating strategy for considered monocyte (C,D) and lymphocyte (E–H) subpopulations. (A) Selection of alive cells after Ficoll gradient; (B) morphological identification of monocytes and lymphocytes; (C) HLA DR+ monocytes; (D) CD14+ CD16+ and CD 14++ CD16− HLA DR+ monocytes; (E) T-helper (CD4+) lymphocytes; (F) cytotoxic (CD8+) lymphocytes; (G) B lymphocytes; (H) natural killer (NK) lymphocytes. [file Image_1.tiff]
